# Community-based football in men with prostate cancer: 1-year follow-up on a pragmatic, multicentre randomised controlled trial

**DOI:** 10.1371/journal.pmed.1002936

**Published:** 2019-10-01

**Authors:** Eik Dybboe Bjerre, Thomas Hindborg Petersen, Anders Bojer Jørgensen, Christoffer Johansen, Peter Krustrup, Bente Langdahl, Mads Hvid Poulsen, Søren Sørensen Madsen, Peter Busch Østergren, Michael Borre, Mikael Rørth, Klaus Brasso, Julie Midtgaard

**Affiliations:** 1 University Hospitals’ Centre for Health Research, Rigshospitalet, Copenhagen, Denmark; 2 Unit of Survivorship, Danish Cancer Society Research Center, Copenhagen, Denmark; 3 Department of Sports Science and Clinical Biomechanics, University of Southern Denmark, Odense, Denmark; 4 Sport and Health Sciences, College of Life and Environmental Sciences, University of Exeter, Exeter, United Kingdom; 5 Department of Endocrinology and Internal Medicine, Aarhus University Hospital, Aarhus, Denmark; 6 Department of Urology, Odense University Hospital, Odense, Denmark; 7 Academy of Geriatric Cancer Research, Odense University Hospital, Odense, Denmark; 8 Department of Urology, Hospital of Southwest Denmark/Esbjerg, Esbjerg, Denmark; 9 Department of Urology, Herlev and Gentofte University Hospital, Herlev, Denmark; 10 Department of Urology, Aarhus University Hospital, Aarhus, Denmark; 11 Department of Oncology, Rigshospitalet, University of Copenhagen, Copenhagen, Denmark; 12 Copenhagen Prostate Cancer Center, Department of Urology, Rigshospitalet, University of Copenhagen, Copenhagen, Denmark; 13 Department of Public Health, University of Copenhagen, Copenhagen, Denmark; Curtin University, AUSTRALIA

## Abstract

**Background:**

Physical exercise has been shown to be effective in relation to fatigue, aerobic fitness, and lower body strength in men with prostate cancer. However, research into the clinically relevant effects of interventions conducted in heterogeneous patient populations and in real-life clinical practice settings is warranted.

**Methods and findings:**

We conducted a pragmatic, multicentre, parallel randomised controlled trial in 5 Danish urological departments. Recruitment began in May 2015, the first participant was randomised in June 2015, and the last participant was included in February 2017. In total, 214 men with prostate cancer were randomly assigned to either 6 months of free-of-charge football training twice weekly at a local club (football group [FG]) (*n* = 109) or usual care (usual care group [UG]) (*n* = 105), including brief information on physical activity recommendations at randomisation. Participants were on average 68.4 (SD 6.2) years old, 157 (73%) were retired, 87 (41%) were on castration-based treatment, 19 (9%) had received chemotherapy, and 41 (19%) had skeletal metastases at baseline. In this 1-year follow-up study, we evaluated the effects of community-based football training on the following outcomes: primary outcome, quality of life; secondary outcomes: continuation of football after 6 months, hip and lumbar spine bone mineral density (BMD), mental health score, fat and lean body mass, and safety outcomes, i.e., fractures, falls, and hospital admissions. Intention to treat (ITT) and per protocol (PP) analyses were conducted. No statistically significant between-group difference was observed in change in prostate-cancer-specific quality of life (ITT: 1.9 points [95% CI −1.9 to 5.8], *p* = 0.325; PP: 3.6 points [95% CI −0.9 to 8.2], *p* = 0.119). A statistically significant between-group difference was observed in change in total hip BMD, in favour of FG (0.007 g/cm^2^ [95% CI 0.004 to 0.013], *p* = 0.037). No differences were observed in change in lumbar spine BMD or lean body mass. Among patients allocated to football, 59% chose to continue playing football after the end of the 6-month intervention period. At 1-year follow-up in the PP population, FG participants had more improvement on the Mental Component Summary (2.9 [95% CI 0.0 to 5.7], *p* = 0.048 points higher) than UG participants, as well as a greater loss of fat mass (−0.9 kg [95% CI −1.7 to −0.1], *p* = 0.029). There were no differences between groups in relation to fractures or falls. Hospital admissions were more frequent in UG compared to FG (33 versus 20; the odds ratio based on PP analysis was 0.34 for FG compared to UG). There were 3 deaths in FG and 4 in UG. Main limitations of the study were the physically active control group and assessment of physical activity by means of self-report.

**Conclusions:**

In this trial, participants allocated to football appeared to have improved hip BMD and fewer hospital admissions. Men who played football more than once a week for 1 year lost fat mass and reported improved mental health. Community-based football proved to be acceptable, even when club membership was not subsidised.

**Trial registration:**

ClinicalTrials.gov NCT02430792.

## Abstract 2

Eik Dybboe Bjerre and colleagues reveal the health benefits, including increased bone mineral density, to men with prostate cancer when they engage in sport.

## Author summary

**Why was this study done?:**

- Sports is often referred to as an applicable and useful public health strategy for promoting physical activity in clinical populations, but to our knowledge no randomised controlled trials have examined this possibility in sports clubs.
- Prostate cancer is the most common cancer in men. Men with prostate cancer can suffer severe morbidity from medical treatments that possibly can be mitigated by exercising and being physical active.

**What did the researchers do and find?:**

- We carried out a pragmatic randomised controlled trial involving 214 men with prostate cancer. Participants were either offered football training in a local club or recommended to continue their active normal life, and were studied over 1 year.
- We found that more than half of participants allocated to football training continued with football in the club even when they had to pay their club membership fee themselves.
- In this setting, men with prostate cancer appeared to have improved hip bone density and were also admitted to hospital less frequently when allocated to a local football club. Playing football did not change men’s scores of prostate-cancer-specific quality of life. The men who played football more than 1 time a week had improved mental health scores and lost fat mass.

**What do these findings mean?:**

- Clinicians can encourage men with prostate cancer to engage in local sports activities to mitigate some of the negative effects of medical treatments.
- Sports can be a strategy for some men with prostate cancer in substitution for other exercise.
- Our findings are limited by the setting the study was implemented in: Denmark is a country with a highly active population and many local football clubs.

## Introduction

The number of men with prostate cancer is increasing; an estimated 5.6 million men are living with the disease worldwide, and in Europe, prostate cancer is the leading cause of disability due to cancer [1]. Treatment of the disease, in addition to the disease itself, is a cause of morbidity, including decrease in bone mineral density (BMD), increase in fat mass, and increased risk of type 2 diabetes mellitus and cardiovascular disease, as well as reduced quality of life [2]. Exercise has been shown to mitigate some of these negative side effects, and physical activity has been shown to be associated with increased survival [3–5]. In addition, exercise is also suggested to mitigate emotional lability and depression [6]. Exercise, predominately supervised aerobic and resistance training, has been examined in men with prostate cancer, and 5 trials have reported outcomes after 1 year of exercising [7–11]. Broadly, the interventions entailed whole-body resistance training with loads of 6–12 repetition maximum and aerobic exercise at 60%–85% of heart rate maximum; 2 interventions included impact loading. Overall findings from these trials showed supervised exercise to be safe and efficacious in relation to fatigue, aerobic fitness, and lower body strength [5,7–11]. However, long-term exercise adherence and easy accessibility is a concern [12]. It has been proposed that structures in the local community outside the traditional healthcare system should be used to facilitate the implementation and upscaling of exercise interventions [13]. Based on this background, we initiated the “FC Prostate” research programme; qualitative findings indicated that participants regarded football as an opportunity to regain control and acquire a sense of responsibility for their own health instead of assuming a passive patient role [14]. Furthermore, our single-centre, phase II trial showed significant improvement in hip BMD after 32 weeks [15]. To support knowledge on implementation and generalisability, we subsequently launched FC Prostate Community, a multicentre, phase III, pragmatic trial that initially allocated participants to 6 months of free-of-charge football training at a local club and thereafter gave the football participants the opportunity to continue the intervention by joining the club and paying the membership fee.

The aim was to explore whether patients with prostate cancer accepted exercise in a local football club under regular conditions (i.e., continued to play football after 6 months including paying membership fee), and to examine the potential effects of 1 year of community-based football training in terms of both all randomised participants and just those adhering to the per protocol (PP) criterion of playing football.

## Methods

### Study design, participants, and randomisation

The study was a 2-arm, multicentre randomised controlled trial with a 1:1 allocation to either the football group (FG) or the usual care group (UG). The study was planned with a 6-month intervention period and a 6-month follow-up period. The FC Prostate Community trial adheres to the Consolidated Standards of Reporting Trials (CONSORT) guidelines (S1 CONSORT checklist). Patients were recruited from 5 Danish urological departments. Detailed information on the design, randomisation, and participants has been reported [16] (see S1 Trial protocol). In brief, participants were eligible when diagnosed with prostate cancer regardless of treatment and disease status; only men with osteoporosis (i.e., T-score < 2.5) were excluded. Randomisation was done with a list of random generated numbers with varying block sizes (*n* = 4–8) stratified for centre and treatment (receiving androgen deprivation therapy [ADT] or not) generated by a statistician not in other ways involved in the trial. The trial personnel, who randomised participants, were blinded towards the allocation list using a web-based trial management platform.

Sample size for this trial was based on a detection of a 6-point change in prostate-cancer-specific quality of life (Pca-QoL) measured with the Functional Assessment of Cancer Therapy–Prostate (FACT-P) questionnaire at 12 weeks. The expected standard deviation was 15 FACT-P points, and a 2-sided significance level of 5% and a power of 80% were chosen. Consequently the trial required a minimum of 100 participants in each arm (for further information, see protocol [16]). The trial was conducted in accordance with the Helsinki Declaration following ethical approval by the local ethics committee (file number H-2-2014-099). All participants gave written informed consent before initiating any trial activities. The trial was registered at ClinicalTrials.gov (NCT02430792), and we have previously reported the outcomes at 12 weeks and 6 months in full compliance with the registration [17].

### Intervention

Participants were informed about physical activity guidelines at randomisation. FG participants were invited to play football for 1 hour twice a week at a local football club. The football sessions were planned to comprise a 20-minute warm-up based on the FIFA 11+ program [18], with modified exercises for the upper body, followed by a 20-minute period with drills and lastly a 20-minute period of match play. After 6 months, participants were invited to continue the intervention by joining the club on the local club’s terms, e.g., by paying the membership fee. At randomisation, UG participants took part in a 15- to 30-minute telephone session covering their options for physical activity and free rehabilitation delivered by local municipalities. They did not receive any other offers during the 1-year period [16].

### Outcomes

This 1-year follow-up report presents results on the primary outcome, Pca-QoL, and secondary outcomes: continuation of football after 6 months, hip and lumbar spine BMD, mental health score, fat and lean body mass, and safety outcomes, i.e., fractures, falls, and hospital admissions. Changes in hip and spine BMD and in lean and fat body mass were assessed with dual-energy X-ray absorptiometry (DXA). DXA assessors were blinded to participants’ group allocation. Patient-reported outcomes were Pca-QoL, measured with the FACT-P questionnaire [19]; mental health, assessed by the Mental Component Summary of Short Form-12 together with the individual outcomes of the vitality, social function, role-emotional, and mental health scales [20]; and physical activity, measured with the short International Physical Activity Questionnaire (IPAQ) [21]. Safety outcomes were numbers of fractures and falls requiring medical assessment and hospital admissions. Safety outcomes were assessed through self-report from participants and review of central medical records (from all Danish hospitals). The patient-reported outcomes were obtained using a web-based data capture system.

We also report the number of FG participants who continued playing football after the 6-month intervention, defined as attending more than 5 training sessions, as well as the number who attended 50% or more of the training sessions during the 1-year period.

Outcomes were assessed at 12 weeks, 6 months, and 1 year. The 12-week and 6-month outcome results have been reported elsewhere [17].

### Data analysis

We report 2 types of analysis, one on the intention to treat (ITT) population, i.e., all participants randomised at baseline, and the other on the PP population, i.e., including only those FG participants who adhered to the a priori PP attendance rate of a minimum of 50% of training sessions [16]. The ITT population was analysed using the same statistical strategy described in our protocol for the 12-week and 6-month data [17]. The analyses on the PP population were performed with the use of principles proposed by Hernán and colleagues [22,23]. We adjusted these analyses for prognostic risk factors known at baseline. We used the prognostic risk factors to adjust for the confounding that cannot be stratified for and that may arise when only a subgroup of participants (PP population) is analysed.

For ITT analyses of continuous outcomes, we compared change scores between allocation groups using analysis of covariance, where we included the allocation group and adjusted for age and the stratification factor ADT. Between-group differences are presented as marginal mean differences between allocation groups with 95% confidence intervals and *p*-values. For binary outcomes, we used the chi-squared test to compare differences in proportions. PP analyses on continuous outcomes included allocation group and adjusted for the following baseline values in the model: age, smoking, alcohol consumption, employment, education, marital status, disease stage, tumour grade (Gleason score), prostate cancer treatment, and co-morbidities. For binary outcomes, we used logistic regression models to allow for adjustment of the same variables list as in the analyses of continuous outcomes. All figures show results from reported models. For all models, we checked the distribution of continuous variables for normality, and residuals were scrutinised to validate models. No imputation of missing data was performed as we had low attrition and most missing data were related to death or progression of disease, which were equally distributed between groups. All analyses were done with STATA 15 version 1.

## Results

**Fig 1:**
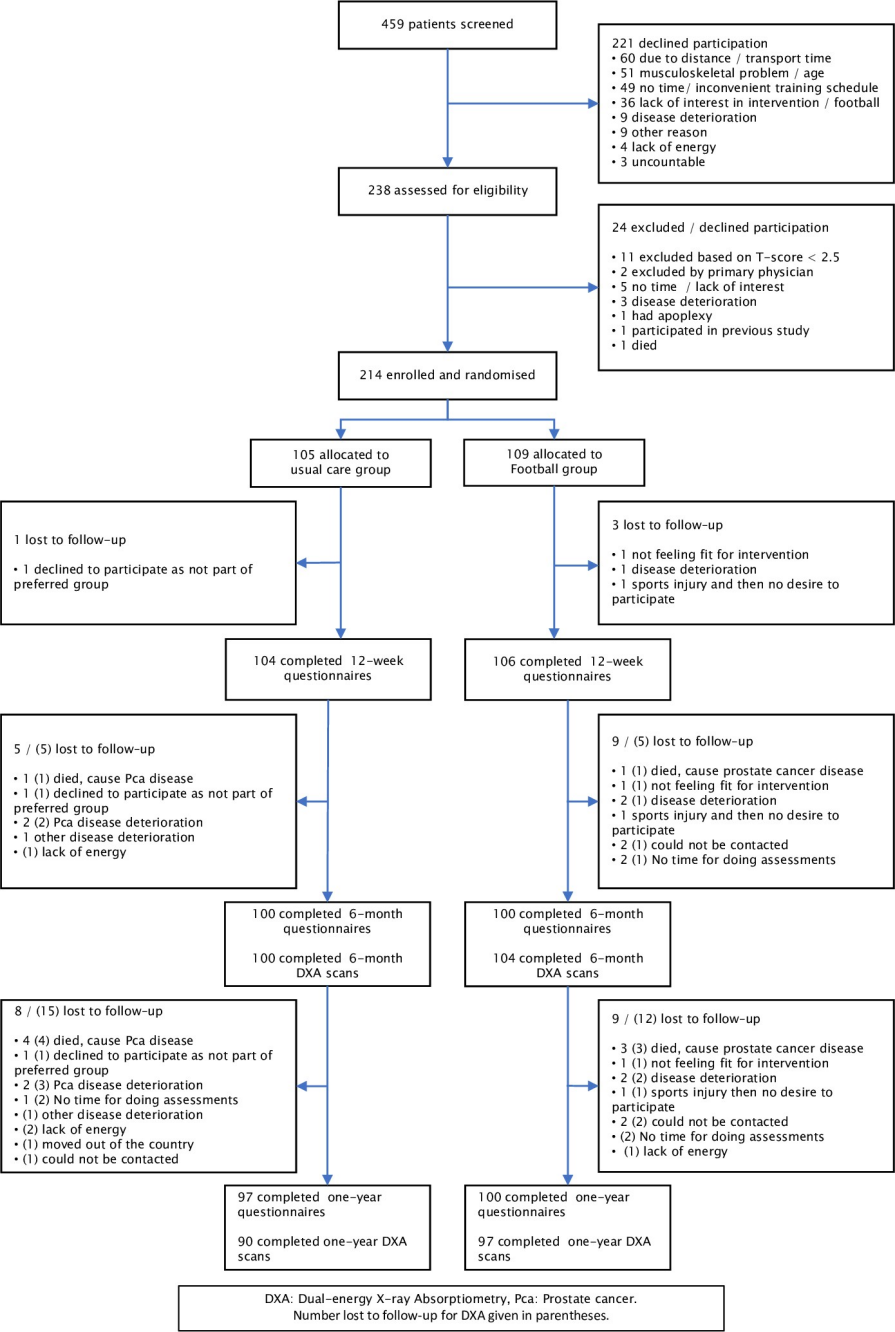
CONSORT 2010 flow diagram.

From June 2015 to February 2017, 214 men were randomised to either FG (*n* = 109) or UG (*n* = 105). Retention, similar in the 2 arms of the study, was 95% at the end of the 6-month intervention period and 92% at 1 year for patient-reported outcomes (*n* = 197) and 87% at 1 year for DXA outcomes (*n* = 187) (Fig 1). Sixty-four FG participants (59%) chose to continue playing football after completion of the 6-month intervention, 78% of whom (*n* = 50) attended ≥50% of the training sessions during the 1-year period. No significant differences existed between FG and UG at baseline (Table 1). Comparing FG participants who attended ≥50% of the training sessions to the ones who attended <50% showed no differences in the outcome variables at baseline, but the groups differed with regard to marital status and number of co-morbidities (S1 Table). During the conduct of the trial, 616 football sessions were held across sites. On average, sessions took 58.8 (58.4–59.3) minutes, with 20.4 (20.0–20.8) minutes spent on warm-up, 16.1 (15.5–16.6) minutes on drills, and 22.3 (21.8–22.9) minutes on match play.

**Table 1:** Baseline characteristics of patients according to allocation group.

	Usual care group (*n* = 105)	Football group		Total (*n* = 214)
		Allocated to football group (*n* = 109)	Played football (*n* = 50)	
Age (years)	69.0 (6.2)	67.8 (6.2)	67.5 (5.9)	68.4 (6.2)
Employment status				
Paid work	26 (25%)	26 (24%)	11 (22%)	52 (24%)
Unemployed	0 (0%)	2 (2%)	1 (2%)	2 (1%)
Sick leave	2 (2%)	1 (1%)	0 (0%)	3 (1%)
Retired	77 (73%)	80 (73%)	38 (76%)	157 (73%)
Education				
No completed education	5 (5%)	7 (6%)	2 (4%)	12 (6%)
Primary education (9th/10th grade)	5 (5%)	4 (4%)	2 (4%)	9 (4%)
Vocational education	28 (27%)	33 (30%)	13 (26%)	61 (29%)
Secondary education (12th grade)	15 (14%)	10 (9%)	6 (12%)	25 (12%)
Completed college or higher	52 (50%)	55 (50%)	27 (54%)	107 (50%)
Marital status				
Married or living with partner	93 (89%)	92 (84%)	48 (96%)	185 (86%)
Other (single, divorced, or widowed)	12 (11%)	17 (16%)	2 (4%)	29 (14%)
Smoking				
Yes	11 (10%)	17 (16%)	5 (10%)	28 (13%)
No, stopped	51 (49%)	49 (45%)	24 (48%)	100 (47%)
No, never	43 (41%)	43 (39%)	21 (42%)	86 (40%)
Alcohol consumption (units of alcohol per week)	8.5 (7.0)	9.1 (7.2)	8.8 (8.0)	8.8 (7.1)
Disease stage				
Localised, prostatectomised	15 (14%)	16 (15%)	9 (18%)	31 (14%)
Localised, not prostatectomised	28 (27%)	27 (25%)	14 (28%)	55 (26%)
Locally advanced	42 (40%)	39 (36%)	17 (34%)	81 (38%)
Metastatic	19 (18%)	26 (24%)	10 (20%)	45 (21%)
Unknown	1 (1%)	1 (1%)	0 (0%)	2 (1%)
ISUP Gleason grading				
Group 1 (Gleason score 2–6)	13 (12%)	15 (14%)	8 (16%)	28 (13%)
Group 2 (Gleason score 3 + 4)	36 (34%)	29 (27%)	17 (34%)	65 (30%)
Group 3 (Gleason score 4 + 3)	13 (12%)	18 (17%)	6 (12%)	31 (14%)
Group 4 (Gleason score 8)	13 (12%)	18 (17%)	9 (18%)	31 (14%)
Group 5 (Gleason score 9–10)	24 (23%)	28 (26%)	10 (20%)	52 (24%)
Unknown	6 (6%)	1 (1%)	0 (0%)	7 (3%)
Number of men with bone metastasis	19 (18%)	22 (20%)	7 (14%)	41 (19%)
Current treatment at baseline				
No treatment (watchful waiting, active surveillance, or previous prostatectomy or radiation)	42 (40%)	46 (42%)	24 (48%)	88 (41%)
Anti-androgen monotherapy	21 (20%)	15 (14%)	7 (14%)	36 (17%)
Castration (surgical or pharmacological)	41 (39%)	46 (42%)	19 (38%)	87 (41%)
Unknown	1 (1%)	2 (2%)	0 (0%)	3 (1%)
Previous treatment at baseline				
Prostatectomy	39 (37%)	27 (25%)	14 (28%)	66 (31%)
Radiation	29 (28%)	37 (34%)	16 (32%)	66 (31%)
ADT and radiation with curative intent	16 (15%)	21 (19%)	9 (18%)	37 (17%)
Chemotherapy (docetaxel)	10 (10%)	9 (8%)	4 (8%)	19 (9%)
No prior or current treatment	24 (22%)	21 (20%)	13 (26%)	45 (21%)
Number of co-morbidities				
0	36 (34%)	28 (26%)	11 (22%)	64 (30%)
1	41 (39%)	38 (35%)	24 (48%)	79 (37%)
2	16 (15%)	30 (28%)	13 (26%)	46 (22%)
3 or more	12 (11%)	13 (12%)	2 (4%)	25 (12%)
Baseline values on outcomes				
Prostate-cancer-specific quality of life (FACT-P, points)	124.6 (16.6)	123.7 (17.3)	124.8 (16.9)	124.1 (16.9)
Mental Component Summary (Short Form-12, points)	52.9 (7.8)	52.8 (6.6)	52.8 (6.2)	52.9 (7.2)
Lean body mass (kg)	57.5 (7.1)	56.6 (6.3)	56.6 (5.5)	57.0 (6.7)
Fat mass (kg)	28.3 (8.9)	27.5 (8.0)	26.8 (8.3)	27.9 (8.4)
Total hip BMD (g/cm^2^)	1.025 (0.138)	1.015 (0.132)	1.005 (0.132)	1.020 (0.134)
Lumbar BMD (g/cm^2^)	1.189 (0.223)	1.188 (0.226)	1.177 (0.216)	1.188 (0.224)
Weekly self-reported physical activity (median MET)^a^	4,098 (2,394–7,732)	3,649 (1,824–6,693)	4,686 (2,631–6,786)	4,046 (2,010–6,845)

Data are mean (standard deviation), *n* (%), or median (interquartile range).

^a^102 patients in the football group and 96 patients in the usual care group.

ADT, androgen deprivation therapy; BMD, bone mineral density; FACT-P, Functional Assessment of Cancer Therapy–Prostate; ISUP, International Society of Urological Pathology; MET, metabolic equivalent of task.

### DXA outcomes

**Fig 2:**
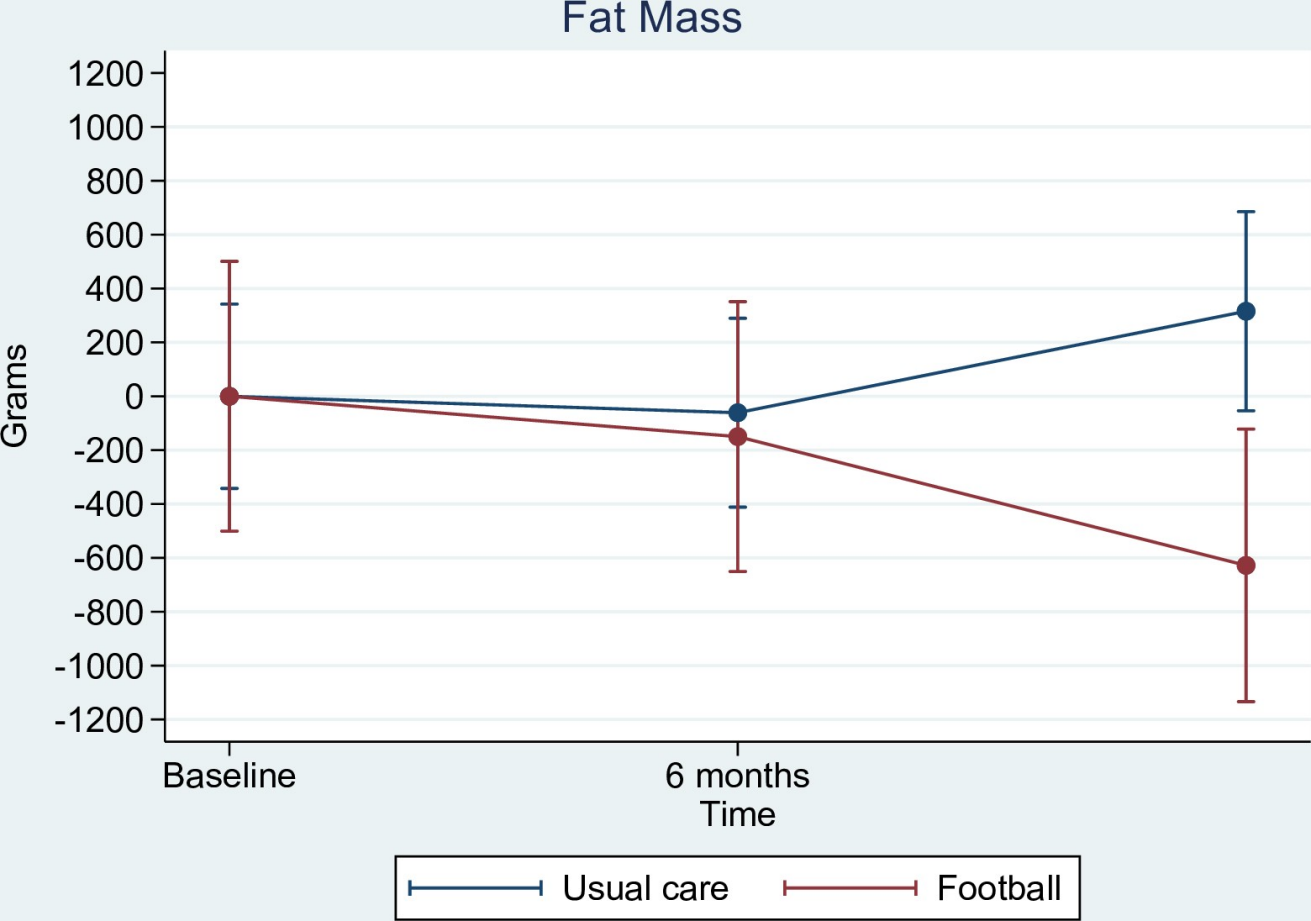
Fat mass changes for per protocol football participants compared to usual care participants.

A statistically significant between-group difference in favour of FG was observed in change in total hip BMD in the ITT population (0.007 g/cm^2^ [95% CI 0.004 to 0.013], *p* = 0.037) and the PP population (0.007 g/cm^2^ [95% CI 0.000 to 0.015], *p* = 0.046) (Tables 2 and 3). Change in spine BMD did not show any statistical difference between groups in the ITT population (0.008 g/cm^2^ [95% CI −0.005 to 0.022], *p* = 0.215) or the PP population (0.003 g/cm^2^ [95% CI −0.012 to 0.019], *p* = 0.673). No between-group differences were observed in change in lean body mass in either the ITT population (−0.2 kg [95% CI −0.7 to 0.2], *p* = 0.339) or the PP population (0.2 kg [95% CI −0.3 to 0.7], *p* = 0.506). The between-group difference in change in fat mass was −0.6 kg (95% CI −1.3 to 0.1, *p* = 0.108) in the ITT analysis and −0.9 kg (95% CI −1.7 to −0.1, *p* = 0.029) in the PP analysis, i.e., FG participants lost more fat mass than UG participants after 1 year (Fig 2).

**Table 2:** Outcomes at 1 year based on intention to treat population.

Outcome	Usual care group		Football group		Effectiveness analysis: Difference between groups adjusted for ADT, age, and baseline score	
	*n*	Mean (95% CI)	*N*	Mean (95% CI)	Mean (95% CI)	*p-*Value
Change in lean body mass (kg)	90	−0.1 (−0.4 to 0.3)	97	−0.3 (−0.6 to 0.0)	−0.2 (−0.7 to 0.2)	0.339
Change in fat mass (kg)	90	0.3 (−0.2 to 0.8)	97	−0.3 (−0.8 to 0.2)	−0.6 (−1.3 to 0.1)	0.108
Change in total hip bone mineral density (g/cm^2^)	90	0.001 (−0.003 to 0.006)	97	0.008 (0.003 to 0.012)	0.007 (0.004 to 0.013)	0.037
Change in lumbar spine L1–L4 bone mineral density (g/cm^2^)	89	0.009 (−0.000 to 0.018)	96	0.017 (0.008 to 0.027)	0.008 (−0.005 to 0.022)	0.215
Change in prostate-cancer-specific quality of life (FACT-P total score, higher is better)	97	−4.2 (−6.9 to −1.4)	100	−2.2 (−4.9 to 0.5)	1.9 (−1.9 to 5.8)	0.325
Change in mental health scale (SF-12, higher is better)	97	−2.1 (−3.8 to −0.4)	100	0.2 (−1.4 to 1.9)	2.3 (−0.1 to 4.7)	0.055
Change in Mental Component Summary (SF-12, higher is better)	97	−1.9 (−3.5 to −0.4)	100	0.2 (−1.3 to 1.8)	2.2 (−0.0 to 4.4)	0.054

FACT-P, Functional Assessment of Cancer Therapy–Prostate; SF-12, Short Form-12.

**Table 3:** Outcomes at 1 year based on per protocol population.

Outcome	Usual care group		Played football		Efficacy analysis: Difference between groups adjusted for risk variables*	
	*n*	Mean (95% CI)	*n*	Mean (95% CI)	Mean (95% CI)	*p-*Value
Change in lean body mass (kg)	90	−0.2 (−0.5 to 0.2)	48	0.0 (−0.4 to 0.4)	0.2 (−0.3 to 0.7)	0.506
Change in fat mass (kg)	90	0.3 (−0.2 to 0.8)	50	−0.6 (−1.3 to 0.0)	−0.9 (−1.7 to −0.1)	0.029
Change in total hip bone mineral density (g/cm^2^)	90	0.001 (−0.003 to 0.005)	50	0.008 (0.003 to 0.014)	0.007 (0.000 to 0.015)	0.046
Change in lumbar spine L1–L4 bone mineral density (g/cm^2^)	89	0.012 (0.002 to 0.021)	48	0.015 (0.003 to 0.027)	0.003 (−0.012 to 0.019)	0.673
Change in prostate-cancer-specific quality of life (FACT-P total score, higher is better)	97	−5.0 (−7.6 to −2.3)	50	−1.3 (−5.0 to 2.3)	3.6 (−0.9 to 8.2)	0.119
Change in mental health scale (SF-12, higher is better)	97	−1.8 (−3.6 to −0.1)	49	1.7 (−0.7 to 4.1)	3.5 (0.6 to 6.5)	0.020
Change in Mental Component Summary (SF-12, higher is better)	97	−1.8 (−3.5 to −0.2)	49	1.0 (−1.2 to 3.3)	2.9 (0.0 to 5.7)	0.048

*Risk variables used to adjust for confounding when analysing efficacy: baseline value, age, smoking, alcohol consumption, employment status, education, marital status, disease stage, Gleason score, treatment, and co-morbidities.

FACT-P, Functional Assessment of Cancer Therapy–Prostate; SF-12, Short Form-12.

### Patient-reported outcomes

For the both the ITT and PP population, Pca-QoL showed no statistical significant difference between groups (change in ITT population: 1.9 points [95% CI −1.9 to 5.8], *p* = 0.325; change in PP population: 3.6 points [95% CI −0.9 to 8.2], *p* = 0.119). In the ITT population, changes in mental health—both the Mental Component Summary score and the specific mental health scale score—were non-statistically higher in FG than UG (summary: 2.2 points [95% CI −0.0 to 4.4], *p* = 0.054; subscale: 2.3 points [95% CI −0.1 to 4.7], *p* = 0.055).

In the PP population, reported mental health was significantly more improved in FG than UG over 1 year, both on the Mental Component Summary and the specific mental health scale (summary: 2.9 points [95% CI 0.0 to 5.7], *p* = 0.048; subscale: 3.5 points [95% CI 0.6 to 6.5], *p* = 0.020). Subdomains for patient-reported outcomes are reported in S2 Table (ITT analysis) and S3 Table (PP analysis).

### Safety, physical activity, and health-specific outcomes

For the ITT population, the overall number of falls was 13 in FG and 10 in UG, with 1 participant in each group reporting 2 falls (*p* = 0.699). Two fractures were reported in each group. The corresponding odds ratio for sustaining a fracture when playing football at least 1 time a week (PP analysis) was 2.58 (*p* = 0.398) for FG compared to UG.

We found no significant differences between groups with respect to self-reported physical activity behaviour; both groups reported higher metabolic equivalent of task (MET) values at 1 year than at baseline, with a median increase of 387 (IQR 3,539) for FG and 589 (IQR 5,899) for UG.

The number of hospital admissions was significantly different between groups in the ITT population, with 20 in FG and 33 in UG (*p* = 0.016). The corresponding odds ratio for the risk of having a hospital admission when playing football at least 1 time a week (PP analysis) was 0.34 (*p* = 0.042) for FG compared to UG (Tables 4 and 5).

**Table 4:** Safety outcomes based on ITT population.

Safety outcome	Usual care group (*n* = 105)	Football group (*n* = 109)	*p-*Value
Falls			
1	8	11	
2	1	1	
Total	10	13	0.699
Fractures	2	2	0.966
Hospital admissions			
1	12	17	
2	9	0	
3	1	1	
Total	33	20	0.016

ITT, intention to treat.

**Table 5:** Safety outcomes based on PP population.

Safety outcome	Usual care group (*n* = 105)	Played football (*n* = 50)	Odds ratio, *p-*Value
Falls			
1	8	7	
2	1	0	1.36, *p* = 0.602
Fractures	2	2	2.58, *p* = 0.398
Hospital admissions			
1	12	6	
2	9	0	
3	1	0	
Total	33	6	0.34, *p* = 0.042

PP, per protocol.

## Discussion

In this 1-year follow-up study on the effects of community-based football training, more than half of the participants initially randomised to playing football opted to continue playing football at their own expense after completion of the 6-month intervention period. Participants allocated to FG did not report the same decline in quality of life apparent in UG, and their mental health improved. Being in FG improved total hip BMD, and playing football regularly was associated with a 0.9-kg decrease in fat mass. There was no difference between the groups in regard to falls and fractures; a lower number of all-cause hospital admissions was observed in FG, when analysing both the ITT population and the PP population.

The findings of this 1-year follow-up are in most outcomes consistent with the short-term report on the time points 3 and 6 months [17]. However, differences are seen in hip BMD, Mental Component Summary, and hospital admissions, as well as in fat mass changes in PP analyses. The small improvement seen in hip BMD at 1 year, but not at earlier time points, is consistent with the finding in our first smaller explanatory phase II trial, where hip BMD improvements also first were seen at that trial’s 32-week time point [15]. As the process of bone remodelling is slow, it is recognised in radiologist guidelines that BMD changes at the earliest should be evaluated after 6 months when looking at high potent interventions [24]. Moreover, the guidelines regard exercise and other lifestyle interventions as having a low-to-moderate potent effect, thus requiring even longer follow-up times [24]. In addition, the fact that the change is found only in hip BMD and not spine is consistent with the biomechanical loading pattern of football [25]. Previously, at 6 months, statistically significant differences between groups were observed in the Mental Component Summary both based on ITT and PP analyses [17]. In this 1-year report, it is only in the PP analysis that this effect is seen. A possible explanation for this difference is that 41% of participants randomised to FG did not continue with the football intervention, but were still included in the ITT analyses. In support of this explanation, it should be noted that the magnitude of the difference in the Mental Component Summary (i.e., 2.9 points) reported in this follow-up in the PP analysis is similar to that at the 6-month time point (ITT, 2.7 points; PP, 2.5 points). The number of hospital admissions was not statistically significantly different between groups after 6 months, as FG had 11 and UG had 22 (*p* = 0.12) [17]. We believe that the reason why this outcome reached significance after 12 months has to do with the accumulation of the number of hospital admissions from 6 to 12 months, which increased statistical power. Lastly, we observed a statistically significant between-group difference in fat mass change in the PP analysis after 1 year but not after 6 months. This may also be explained by an accumulated effect over time in the men actively engaging in the intervention (for more than 6 months), whereas the men in the control group and the men in FG who did not adhere to football longer term (>6 months) did not lose fat.

To our knowledge, football training is the first type of exercise intervention in men with prostate cancer to show improvement in hip BMD. Our small-scale, single-centre randomised controlled trial, conducted in 2012–2013, previously showed improved hip strength in men with prostate cancer on ADT at 32 weeks [15], and this confirmatory multicentre study corroborates this finding in a larger and more heterogenic population and a more real-world setting. Winters-Stone et al. examined resistance and impact loading and found no skeletal improvement in hip BMD [9]. One possible explanation for the discrepancy between the findings of that study and ours is that impact loading from football varies constantly, and participants have the ability to adjust naturally in response to the intensity of accelerations and decelerations [26]. One of the possible consequences or trade-offs of higher intensity is a greater risk of bone fractures. Although not significant, FG participants had a higher odds ratio for sustaining fractures; however, overall fractures were equally distributed among the allocation groups. The men attending ≥50% of sessions over 1 year decreased their fat mass by 0.9 kg regardless of treatment (ADT or non-ADT). In this regard it is worth noting that, while exercise is already accepted as a strategy to mitigate treatment-induced increases in fat mass in men undergoing ADT [2], reducing fat mass in men with low-risk prostate cancer may also prove beneficial, as obesity has been associated with progression in patients on active surveillance [27].

Men with prostate cancer are at risk of experiencing psychological distress during the course of their disease [6,28]. In a previous study we found that men experienced football as an opportunity to regain control [14], which is why the improved mental health finding, also confirmed by the 6-month report [17], was anticipated. Furthermore, this finding is in line with the results from a recently published trial by Taaffe et al., who found that supervised group-based exercise positively affected vitality and fatigue [10]. In another exercise trial, however, Galvão et al. did not find any changes in mental health, despite positive effects on cardiorespiratory fitness and physical functioning [8]. The complexity of exercise as a behavioural intervention is one possible explanation for these conflicting findings. Exercise can be performed in many different settings, and it can be individually tailored, to maximise the physiological effect, and/or group-based, to enable peer-to-peer relationships. Individually tailored exercise gives the possibility of specific prescription of exercise modes, volume, and intensity; this, however, is not possible with football as the volume and intensity is auto-regulated by participants. The fact that FG participants had fewer hospital admissions was surprising, We are unable to offer a comprehensive explanation for this finding but propose that the greater level of control and insight the men experienced regarding their own health, combined with the support of their peers, may possibly have been a contributing factor [14]. This finding can be a possible avenue of continued exploration in the next generation of exercise trials in men with prostate cancer focusing on exercise as a primary therapy option [11,29].

Our study has various methodological limitations. The analysis of physical activity behaviour was hampered due to large variation in the sample, which is a known methodological issue concerning the responsiveness of IPAQ when used in clinical trials [30]. If the responsiveness of IPAQ is inaccurate, it reduces the reliability of the physical activity change data. If we look beyond the mentioned validity issue related to physical activity change, another possible limitation is that the sampled participants reported being highly physically active (i.e., above recommendations) already at baseline and adhered to this level throughout the trial. This has most likely resulted in an underestimation of the effect of the intervention as we compared a group (i.e., FG) that most likely substituted their normal exercise with football with a control group that appeared to have continued their normal (high) activity level. However, this being a pragmatic trial, we wanted to include a sample that would engage in the intervention in real life. In this perspective, our results are probably a fair reflection of what can be expected under natural circumstances, and may as such be interpreted as successful. Due to the pragmatic design of the trial, the various features of the heterogenic population (i.e., varied disease and co-morbidity status, as seen in Table 1) need to be scrutinised; the study design optimises generalisability, but effect modification between subgroups can reduce the ability to infer from analyses. As a result, we stratified randomisation on the ADT population and checked if differential effects were present in subgroups, but this was not the case. Furthermore, we adjusted PP analyses for the prognostic risk variables at baseline that would confound these analyses. Another issue related to generalisability is that the intervention was implemented in Denmark, a high-income country with many local amateur sports clubs, enabling a reciprocal partnership between hospitals and the local community, which may not be a viable strategy if this infrastructure is not available.

### Conclusions

The current trial is, to our knowledge, the first pragmatic randomised controlled trial to examine the effects of sports participation in a clinical population. Community-based football proved to be an acceptable intervention, even when participants had to pay the membership fee themselves. In this trial, participants allocated to football appeared to have improved hip BMD and fewer hospital admissions. Furthermore, the men who played more than once a week for 1 year improved their mental health and lost fat mass. Future studies should focus on examining the clinically relevant effects of exercise, while implementation studies should explore the accessibility of exercise tailored to local infrastructures.

## Supporting information

S1 Consort checklist (PDF)

S1 Trial protocol (PDF)

S1 Table Baseline characteristics of patients, according to allocation group and attendance. (PDF)

S2 Table Additional patient-reported outcomes at 1 year based on ITT population. (PDF)

S3 Table Additional patient-reported outcomes at 1 year based on PP population. (PDF)

## Abbrevations

ADT: androgen deprivation therapy
BMD: bone mineral density
DXA: dual-energy X-ray absorptiometry
FACT-P: Functional Assessment of Cancer Therapy–Prostate
FG: football group
IPAQ: International Physical Activity Questionnaire
ITT: intention to treat
Pca-QoL: prostate-cancer-specific quality of life
PP: per protocol
UG: usual care group

## References

[1] Vos T, Abajobir AA, Abate KH, Abbafati C, Abbas KM, Abd-Allah F, et al Global, regional, and national incidence, prevalence, and years lived with disability for 328 diseases and injuries for 195 countries, 1990–2016: a systematic analysis for the Global Burden of Disease Study 2016. Lancet. 2017;390(10100):1211–59. 10.1016/S0140-6736(17)32154-2 28919117 PMC5605509

[2] Nguyen PL, Alibhai SM, Basaria S, D’Amico AV, Kantoff PW, Keating NL, et al Adverse effects of androgen deprivation therapy and strategies to mitigate them. Eur Urol. 2015;67(5):825–36. 10.1016/j.eururo.2014.07.010 25097095

[3] Wang Y, Jacobs EJ, Gapstur SM, Maliniak ML, Gansler T, McCullough ML, et al Recreational physical activity in relation to prostate cancer-specific mortality among men with nonmetastatic prostate cancer. Eur Urol. 2017;72(6):931–9. 10.1016/j.eururo.2017.06.037 28711382

[4] Friedenreich CM, Wang Q, Neilson HK, Kopciuk KA, McGregor SE, Courneya KS. Physical activity and survival after prostate cancer. Eur Urol. 2016;70(4):576–85. 10.1016/j.eururo.2015.12.032 26774959

[5] Bourke L, Smith D, Steed L, Hooper R, Carter A, Catto J, et al Exercise for men with prostate cancer: a systematic review and meta-analysis. Eur Urol. 2016;69(4):693–703. 10.1016/j.eururo.2015.10.047 26632144

[6] Donovan KA, Walker LM, Wassersug RJ, Thompson LM, Robinson JW. Psychological effects of androgen-deprivation therapy on men with prostate cancer and their partners. Cancer. 2015;121(24):4286–99. 10.1002/cncr.29672 26372364

[7] Segal RJ, Reid RD, Courneya KS, Sigal RJ, Kenny GP, Prud’Homme DG, et al Randomized controlled trial of resistance or aerobic exercise in men receiving radiation therapy for prostate cancer. J Clin Oncol. 2009;27(3):344–51. 10.1200/JCO.2007.15.4963 19064985

[8] Galvão DA, Spry N, Denham J, Taaffe DR, Cormie P, Joseph D, et al A multicentre year-long randomised controlled trial of exercise training targeting physical functioning in men with prostate cancer previously treated with androgen suppression and radiation from TROG 03.04 radar. Eur Urol. 2014;65(5):856–64. 10.1016/j.eururo.2013.09.041 24113319

[9] Winters-Stone KM, Dobek JC, Bennett JA, Maddalozzo GF, Ryan CW, Beer TM. Skeletal response to resistance and impact training in prostate cancer survivors. Med Sci Sports Exerc. 2014;46(8):1482–8. 10.1249/MSS.0000000000000265 24500540 PMC4101037

[10] Taaffe DR, Newton RU, Spry N, Joseph D, Chambers SK, Gardiner RA, et al Effects of different exercise modalities on fatigue in prostate cancer patients undergoing androgen deprivation therapy: a year-long randomised controlled trial. Eur Urol. 2017;72(2):293–9. 10.1016/j.eururo.2017.02.019 28249801

[11] Bourke L, Stevenson R, Turner R, Hooper R, Sasieni P, Greasley R, et al Exercise training as a novel primary treatment for localised prostate cancer: a multi-site randomised controlled phase II study. Sci Rep. 2018;8(1):8374 10.1038/s41598-018-26682-0 29849032 PMC5976628

[12] Parsons JK. Prostate cancer and the therapeutic benefits of structured exercise. J Clin Oncol. 2014;32(4):271–2. 10.1200/JCO.2013.53.4289 24344219

[13] Reis RS, Salvo D, Ogilvie D, Lambert EV, Goenka S, Brownson RC. Scaling up physical activity interventions worldwide: stepping up to larger and smarter approaches to get people moving. Lancet. 2016;388(10051):1337–48. 10.1016/S0140-6736(16)30728-0 27475273 PMC5193005

[14] Bruun DM, Krustrup P, Hornstrup T, Uth J, Brasso K, Rorth M, et al “All boys and men can play football”: a qualitative investigation of recreational football in prostate cancer patients. Scand J Med Sci Sports. 2014;24(Suppl 1):113–21. 10.1111/sms.12193 24944135

[15] Uth J, Hornstrup T, Christensen JF, Christensen KB, Jorgensen NR, Schmidt JF, et al Efficacy of recreational football on bone health, body composition, and physical functioning in men with prostate cancer undergoing androgen deprivation therapy: 32-week follow-up of the FC Prostate randomised controlled trial. Osteoporos Int. 2016;27(4):1507–18. 10.1007/s00198-015-3399-0 26572756

[16] Bjerre E, Bruun DM, Tolver A, Brasso K, Krustrup P, Johansen C, et al Effectiveness of community-based football compared to usual care in men with prostate cancer: protocol for a randomised, controlled, parallel group, multicenter superiority trial (The FC Prostate Community Trial). BMC Cancer. 2016;16(1):767 10.1186/s12885-016-2805-0 27716218 PMC5048405

[17] Bjerre ED, Brasso K, Jorgensen AB, Petersen TH, Eriksen AR, Tolver A, et al Football compared with usual care in men with prostate cancer (FC Prostate Community Trial): a pragmatic multicentre randomized controlled trial. Sports Med. 2019;49(1):145–58. 10.1007/s40279-018-1031-0 30506427 PMC6349963

[18] Soligard T, Myklebust G, Steffen K, Holme I, Silvers H, Bizzini M, et al Comprehensive warm-up programme to prevent injuries in young female footballers: cluster randomised controlled trial. BMJ. 2008;337:a2469 10.1136/bmj.a2469 19066253 PMC2600961

[19] Esper P, Mo F, Chodak G, Sinner M, Cella D, Pienta KJ. Measuring quality of life in men with prostate cancer using the functional assessment of cancer therapy-prostate instrument. Urology. 1997;50(6):920–8. 10.1016/S0090-4295(97)00459-7 9426724

[20] Gandek B, Ware JE, Aaronson NK, Apolone G, Bjorner JB, Brazier JE, et al Cross-validation of item selection and scoring for the SF-12 Health Survey in nine countries: results from the IQOLA Project. J Clin Epidemiol. 1998;51(11):1171–8. 10.1016/s0895-4356(98)00109-7 9817135

[21] International Physical Activity Questionnaire. IPAQ scoring protocol. 2019 [cited 2019 Jun 28]. Available from: https://sites.google.com/site/theipaq/scoring-protocol.

[22] Hernán MA, Hernandez-Diaz S, Robins JM. Randomized trials analyzed as observational studies. Ann Intern Med. 2013;159(8):560–2. 10.7326/0003-4819-159-8-201310150-00709 24018844 PMC3860874

[23] Hernán MA, Robins JM. Per-protocol analyses of pragmatic trials. N Engl J Med. 2017;377(14):1391–8. 10.1056/NEJMsm1605385 28976864

[24] Siminoski K, O’Keeffe M, Brown JP, Burrell S, Coupland D, Dumont M, et al Canadian Association of Radiologists technical standards for bone mineral densitometry reporting. Can Assoc Radiol J. 2013;64(4):281–94. 10.1016/j.carj.2013.07.006 24314581

[25] Lees A, Nolan L. The biomechanics of soccer: a review. J Sports Sci. 1998;16(3):211–34. 10.1080/026404198366740 9596356

[26] Lanyon LE. Using functional loading to influence bone mass and architecture: objectives, mechanisms, and relationship with estrogen of the mechanically adaptive process in bone. Bone. 1996;18(1 Suppl):37s–43s. 10.1016/8756-3282(95)00378-9 8717546

[27] Bhindi B, Kulkarni GS, Finelli A, Alibhai SM, Hamilton RJ, Toi A, et al Obesity is associated with risk of progression for low-risk prostate cancers managed expectantly. Eur Urol. 2014;66(5):841–8. 10.1016/j.eururo.2014.06.005 24954793

[28] Gao W, Bennett MI, Stark D, Murray S, Higginson IJ. Psychological distress in cancer from survivorship to end of life care: prevalence, associated factors and clinical implications. Eur J Cancer. 2010;46(11):2036–44. 10.1016/j.ejca.2010.03.033 20447824

[29] Galvao DA, Hayne D, Frydenberg M, Chambers SK, Taaffe DR, Spry N, et al Can exercise delay transition to active therapy in men with low-grade prostate cancer? A multicentre randomised controlled trial. BMJ Open. 2018;8(4):e022331 10.1136/bmjopen-2018-022331 29678994 PMC5914709

[30] van Poppel MNM, Chinapaw MJM, Mokkink LB, van Mechelen W, Terwee CB. Physical activity questionnaires for adults. Sports Med. 2010;40(7):565–600. 10.2165/11531930-000000000-00000 20545381

