# Supplementary material for: Community-based football in men with prostate cancer: 1-year follow-up on a pragmatic, multicentre randomised controlled trial
Source: PLoS Med. 2019 Oct 1;16(10):e1002936. doi: 10.1371/journal.pmed.1002936 (PMC6771996; doi:10.1371/journal.pmed.1002936)
Supplement: S1 Table — (PDF) [file pmed.1002936.s003.pdf]

Table 1 Baseline characteristics of patients, according to allocation group and attendance

	Usual care group (n=105)	Allocated to Football group (n=109)	Continued playing football (n=50)	Total (n=214)
Age (years)	69.0 (6.2)	67.8 (6.2)	67.5 (5.9)	68.4 (6.2)
Employment status				
Paid work	26 (25%)	26 (24%)	11 (22%)	52 (24%)
Unemployed	0 (0%)	2 (2%)	1 (2%)	2 (1%)
Sick leave	2 (2%)	1 (1%)	0 (0%)	3 (1%)
Retired	77 (73%)	80 (73%)	38 (76%)	157 (73%)
Education				
No education	5 (5%)	7 (6%)	2 (4%)	12 (6%)
Primary education (9th/10th grade)	5 (5%)	4 (4%)	2 (4%)	9 (4%)
Vocational education	28 (27%)	33 (30%)	13 (26%)	61 (29%)
Secondary education (12th grade)	15 (14%)	10 (9%)	6 (12%)	25 (12%)
Completed college or higher	52 (50%)	55 (50%)	27 (54%)	107 (50%)
Marital status				
Married or living with partner	93 (89 %)	92 (84%)	48 (96%)	185 (86%)
Other (single, divorced, or widowed)	12 (11%)	17 (16%)	2 (4%)	29 (14%)
Smoking status				
Yes	11 (10 %)	17 (16%)	5 (10%)	28 (13%)
No, stopped	51 (49%)	49 (45%)	24 (48%)	100 (47%)
No, never	43 (41%)	43 (39%)	21 (42%)	86 (40%)
Alcohol consumption (units of alcohol per week)	8.5 (7.0)	9.1 (7.2)	8.8 (8.0)	8.8 (7.1)
Risk group				
Localised. prostatectomised	15 (14%)	16 (15%)	9 (18%)	31 (14%)
Localised. not prostatectomised	28 (27%)	27 (25%)	14 (28%)	55 (26 %)
Locally advanced	42 (40%)	39 (36%)	17 (34%)	81 (38 %)
Metastatic	19 (18%)	26 (24%)	10 (20%)	45 (21 %)
Unknown	1 (1%)	1 (1%)	0 (0%)	2 (1%)
ISUP Gleason grading				
Group 1 (Gleason score 2-6)	13 (12%)	15 (14%)	8 (16%)	28 (13 %)
Group 2 (Gleason score 3+4)	36 (34%)	29 (27%)	17 (34%)	65 (30%)
Group 3 (Gleason score 4+3)	13 (12%)	18 (17%)	6 (12%)	31 (14 %)
Group 4 (Gleason score 8)	13 (12%)	18 (17%)	9 (18%)	31 (14 %)
Group 5 (Gleason score 9-10)	24 (23%)	28 (26%)	10 (20%)	52 (24%)
Unknown	6 (6%)	1 (1%)	0 (0%)	7 (3%)
Number of men with bone metastasis	19 (18%)	22 (20%)	7 (14%)	41 (19 %)
Current treatment at baseline				
No treatment (watchful waiting, active surveillance, previous prostatectomy, or radiation)	42 (40%)	46 (42%)	24 (48%)	88 (41%)
Anti-androgen monotherapy	21 (20%)	15 (14%)	7 (14%)	36 (17%)
Castration (surgical or pharmacological)	41 (39%)	46 (42%)	19 (38%)	87 (41%)
Unknown	1 (1%)	2 (2%)	0 (0%)	3 (1%)
Previous treatment at baseline				
Prostatectomy	39 (37%)	27 (25%)	14 (28 %)	66 (31%)
Radiation	29 (28%)	37 (34%)	16 (32 %)	66 (31%)
ADT and radiation with curative intent	16 (15%)	21 (19%)	9 (18 %)	37 (17%)
Chemotherapy (docetaxel)	10 (10%)	9 (8%)	4 (8%)	19 (9%)
No prior or current treatment	24 (22%)	21 (20%)	13 (26 %)	45 (21%)
Number of co-morbidities				
Zero	36 (34%)	28 (26%)	11 (22%)	64 (30%)
One	41 (39%)	38 (35%)	24 (48%)	79 (37%)
Two	16 (15%)	30 (28%)	13 (26%)	46 (22%)
Three or more	12 (11%)	13 (12%)	2 (4%)	25 (12%)
Baseline values on outcomes				
Prostate cancer-specific quality of life (FACT-P, points)	124.6 (16.6)	123.7 (17.3)	124.8 (16.9)	124.1 (16.9)
Mental Component Summary (Short Form-12, points)	52.9 (7.8)	52.8 (6.6)	52.8 (6.2)	52.9 (7.2)
Lean body mass (kg)	57.5 (7.1)	56.6 (6.3)	56.6 (5.5)	57.0 (6.7)
Fat mass (kg)	28.3 (8.9)	27.5 (8.0)	26.8 (8.3)	27.9 (8.4)
Total hip BMD (g/cm^2)	1.025 (0.138)	1.015 (0.132)	1.005 (0.132)	1.020 (0.134)
Lumbar BMD (g/cm^2)	1.189 (0.223)	1.188 (0.226)	1.177 (0.216)	1.188 (0.224)
Weekly self-reported PA (median MET)[a]	4098 (2394–7732)	3649 (1824–6693)	4686 (2631-6786)	4046 (2010–6845)

ISUP = International Society of Urological Pathology; ADT = androgen deprivation therapy; FACT-P = Functional Assessment of Cancer Therapy-Prostate; BMD = bone mineral density. Data are mean (standard deviation), n (%) or median (interquartil range). [A]102 patients in the FG and 96 patients in the UG
