# Supplementary material for: Community-based football in men with prostate cancer: 1-year follow-up on a pragmatic, multicentre randomised controlled trial
Source: PLoS Med. 2019 Oct 1;16(10):e1002936. doi: 10.1371/journal.pmed.1002936 (PMC6771996; doi:10.1371/journal.pmed.1002936)
Supplement: S2 Table — (PDF) [file pmed.1002936.s004.pdf]

S2 Table Additional patient-reported outcomes at one year based on ITT population

	Usual care group		Allocated to football		Effectiveness analyses, difference between groups, mean (95 % CI)	
	n	Mean (95% CI)	n	Mean (95% CI)	Adjusted for ADT, age, and baseline score	p value
Change in FACT-P subscale 1 (higher is better)						
	97	-0.5 (-1.1 to 0.2)	100	-0.4 (-1.0 to 0.3)	0.1 (-0.8 to 1.0)	0.846
Change in FACT-P subscale 2 (higher is better)						
	97	-0.6 (-1.4 to 0.1)	100	0.2 (-0.5 to 1.0)	0.9 (-0.2 to 1.9)	0.105
Change in FACT-P subscale3 (higher is better)						
	97	-0.4 (-1.0 to 0.1)	100	0.1 (-0.4 to 0.7)	0.5 (-0.2 to 1.3)	0.165
Change in FACT-P subscale 4 (higher is better)						
	97	-1.5 (-2.3 to -0.7)	100	-1.0 (-1.7 to -0.2)	0.5 (-0.6 to 1.6)	0.357
Change in FACT-P subscale 5 (higher is better)						
	97	-1.5 (-2.4 to -0.5)	100	-1.2 (-2.2 to -0.3)	0.2 (-1.1 to 1.6)	0.727
Change in vitality (SF-12, higher is better)						
	97	-2.0 (-3.7 to -0.3)	100	-1.0 (-2.7 to 0.6)	1.0 (-1.3 to 3.4)	0.412
Change in social functioning (SF-12, higher is better)						
	97	-1.6 (-2.9 to -0.3)	100	-1.1 (-2.3 to 0.2)	0.5 (-1.2 to 2.4)	0.555
Change in role limitation emotional (SF-12, higher is better)						
	97	-1.8 (-3.5 to -0.0)	100	-0.6 (-2.2 to 1.1)	1.2 (-1.2 to 3.6)	0.334

ITT = intention-to-treat; ADT = androgen deprivation therapy, CI: confidence interval, FACT-P = Functional Assessment of Cancer Therapy-Prostate; SF-12 = Short Form-12.
