# Supplementary material for: Community-based football in men with prostate cancer: 1-year follow-up on a pragmatic, multicentre randomised controlled trial
Source: PLoS Med. 2019 Oct 1;16(10):e1002936. doi: 10.1371/journal.pmed.1002936 (PMC6771996; doi:10.1371/journal.pmed.1002936)
Supplement: S3 Table — (PDF) [file pmed.1002936.s005.pdf]

S3 **Table** Patient-reported outcomes at one year based on PP population

	Usual care group		Played football group		Efficacy analyses, difference between groups, mean (95% CI)	
	n	Mean (95% CI)	n	Mean (95% CI)	Adjusted for risk variables*	p value
Change in FACT-P subscale 1 (higher is better)						
	97	-0.6 (-1.2 to -0.4)	50	-0.4 (-1.2 to 0.3)	0.2 (-0.8 to 1.1)	0.728
Change in FACT-P subscale 2 (higher is better)						
	97	-0.8 (-1.6 to 0.1)	50	0.0 (-1.1 to 1.1)	0.8 (-0.6 to 2.2)	0.274
Change in FACT-P subscale3 (higher is better)						
	97	-0.5 (-1.1 to 0.1)	50	0.4 (-0.4 to 1.2)	0.9 (-0.1 to 1.9)	0.063
Change in FACT-P subscale 4 (higher is better)						
	97	-1.6 (-2.4 to -0.8)	49	-0.6 (-1.7 to 0.5)	1.0 (-0.4 to 2.4)	0.163
Change in FACT-P subscale 5 (higher is better)						
	97	-1.4 (-2.3 to -0.6)	49	-0.8 (-2.0 to 0.3)	0.6 (-0.9 to 2.0)	0.424
Change in vitality (SF-12, higher is better)						
	97	-2.2 (-4.0 to -0.5)	49	-0.7 (-3.0 to 1.6)	1.5 (-1.4 to 4.5)	0.314
Change in social functioning (SF-12, higher is better)						
	97	-1.8 (-3.1 to -0.5)	49	-0.6 (-2.4 to 1.2)	1.2 (-1.1 to 3.4)	0.305
Change in role limitation emotional (SF-12, higher is better)						
	97	-1.6 (-3.3 to 0.2)	49	0.2 (-2.2 to 2.5)	1.7 (-1.3 to 4.7)	0.261

* Risk variables used to adjust for confounding when analysing efficacy: baseline value, age, smoking, alcohol consumption, employment status, education, marital status, disease stage, Gleason score, treatment, and co-morbidities

PP = per-protocol; CI: confidence interval, FACT-P = Functional Assessment of Cancer Therapy-Prostate; SF-12 = Short Form-12.
